# P13. Intra-tumoral and surrogate immune responses in patients treated with the engineered anti-PD-L1 antibody (MPDL3280A)

**DOI:** 10.1186/2051-1426-2-S2-P4

**Published:** 2014-03-12

**Authors:** M Kowanetz, MT Vu, J Wu, H Koeppen, H Kohrt, S Gettinger, C Cruz, M Denker, DS Chen, P Hegde

**Affiliations:** 1 Genentech, Inc., South San Francisco, CA, USA; 2 Stanford University Cancer Institute, Stanford, CA, USA; 3 Yale School of Medicine, New Haven, CT, USA; 4 Vall d'Hebron University Hospital, Barcelona, Spain

## Background

PD-L1 regulates CD8 T cell/Th1 immune responses. PD-L1 expressed in the tumor microenvironment can bind to PD-1 or B7.1 on activated T cells and mediate cancer immune evasion. MPDL3280A is a human mAb containing an engineered Fc-domain designed to optimize efficacy and safety that targets PD-L1 and blocks it from binding to its receptors.

## Methods

Immunologic pharmacodynamics effects were evaluated in tumors and bloods from patients treated with MPDL3280A. MPDL3280A was administered IV q3w in >300 pts with locally advanced or metastatic solid tumors. PD-L1 and CD8 were measured by IHC. PD-L1 expression was evaluated in tumor and intra-tumoral immune cells. CD8 was assessed in the tumor center, periphery and invasive margin. The expression of ≈90 immune-related markers was evaluated at baseline (BL) and on-treatment using a custom-designed immunochip. BL tumor samples were available for 125 pts, and matched on-treatment samples were available for 31 pts. Further, blood-based biomarkers and circulating immune subsets were serially measured in 114 patients by modified ELISA and FACS, respectively.

## Results

On treatment, responding tumors showed increase in expression of tumor cell PD-L1 and infiltration of CD8+ T-cells and a Th1-dominant immune infiltrate, providing evidence for adaptive PD-L1 up-regulation. Non-responders showed minimal tumor CD8+ T-cell infiltration and an absence of T-cell activation (measured by Granzymes, Perforin and EOMES expression). We also profiled circulating biomarkers for their association with clinical outcomes. A sub-population of patients, including those with RCC and melanoma, exhibited elevated PD-L1 expression on circulating T-cells at BL that was associated with response to MPDL3280A. On treatment, we observed a delayed increase in frequency of CD4+ICOS+ and CD4+PD1+ T-cells in patients responding to MPDL3280A monotherapy. In contrast, frequency of CD8+HLA-DR+Ki67+ T-cells increased shortly following the first dose of MPDL3280A and returned to baseline levels by the end of cycle 2 when assessed in all patients, representing a transient pharmacodynamic measurement of PD-L1 inhibition. On-treatment, increase in plasma IL-6 was associated with disease progression. Associations with other circulating markers of inflammation including CRP with clinical outcomes will be presented.

## Conclusions

Our data show that changes in pharmacodynamic immune markers are associated with clinical outcomes to MPDL3280A. These data provide mechanistic insights into immune checkpoint inhibition in cancer and identify potential biomarkers that may be monitored as on-treatment markers of clinical activity for patients treated with inhibitors of PD-L1/PD1 pathway.

